# Spexin Suppress Food Intake in Zebrafish: Evidence from Gene Knockout Study

**DOI:** 10.1038/s41598-017-15138-6

**Published:** 2017-11-07

**Authors:** Binbin Zheng, Shuisheng Li, Yun Liu, Yu Li, Huapu Chen, Haipei Tang, Xiaochun Liu, Haoran Lin, Yong Zhang, Christopher H. K. Cheng

**Affiliations:** 1 State Key Laboratory of Biocontrol, Institute of Aquatic Economic Animals, and the Guangdong Province Key Laboratory for Aquatic Economic Animals, School of Life Sciences, Sun Yat-Sen University, Guangzhou, 510275 China; 2 School of Biomedical Sciences, The Chinese University of Hong Kong, Shatin, New Territories, Hong Kong, China; 3 Present Address: Zhanjiang City State Key Laboratory of Marine Ecology and Environment, Fisheries College,Guangdong Ocean University, Zhanjiang, 524088 China

## Abstract

Spexin1 (SPX1) is a newly discovered neuropeptide in vertebrates. Its biological function remains to be elucidated. In this study, we have generated the zebrafish *spx*1^*−/−*^ mutant lines using transcription activator-like effector nucleases. Phenotypes of the *spx1* ^***−/−***^ mutant zebrafish were analyzed in order to understand the effects on reproduction and food intake. The reproductive capability is not impaired in *spx1* mutant zebrafish. However, we found that the *spx1* ^***−/−***^ mutant fish had a higher food intake than the wild type (WT) fish. Real-time PCR revealed that the expression level of agouti-relate protein 1 (AgRP1), a significant appetite stimulant, was significantly higher in *spx1* ^***−/−***^ mutant fish after feeding. Intracranial administration of SPX1 could also reduce the mRNA expression of the AgRP1. These data suggest that SPX1 might decrease the food intake by down regulating the expression level of *agrp1*. Furthermore, *spx1* ^***−/−***^ mutant fish exhibited higher glucose, triacylglycerol and cholesterol in the serum than WT fish. However, the hyperphagia did not lead to a higher growth rate or body fat percentage. Taken together, our study suggests that SPX1 may serve as a satiety signal molecular by suppressing the AgRP1 in the brain.

## Introduction

Spexin (SPX), also named neuropeptide Q, is a novel neuropeptide predicted by bioinformatics before its purification and identification^1–3^. The prepropeptide of SPX presents a typical secretory protein structure with the 14-amino acid (AA) mature peptide flanked by a hydrophobic signal peptide and two putative prohormone cleavage/amidation sites (RR/GKR)^2^. Notably, the mature peptide NWTPQAMLYLKGAQ and the proteolytic sites are highly conserved across vertebrates^4^, suggesting that SPX might be essential for the survival of species. As revealed by nuclear magnetic resonance spectroscopies, the solution structure of SPX was obtained and the molecular surface of it was highly hydrophobic except for the Lys, which was believed to play a key role to interact with the receptor^5^. Another paralogous SPX gene, termed *SPX2*, was recently identified in non-mammalian vertebrates including chicken, xenopus and zebrafish and it was found that *SPX*s, along with *kisspeptin* (*KISS*) and *galanin* (*GAL*) genes, might probably arouse from the same ancestor. Inspired by this, a ligand-receptor interaction study revealed that SPXs exhibited high potency towards galanin receptor 2/3 and were likely the natural ligands of GalR2/3^6,7^.

The mRNA or protein expression level of SPX in human^3^, rat^8^ and goldfish^4^ was determined in various tissues including brain, liver, kidney, placenta and gonad. Among the endocrine organs mentioned above, the SPX-like immunoreactivity was mainly presented in the epithelia, suggesting its involvement in trans-epithelia transport as a secretary peptide with multiple functions in different tissues^8^. However, research on the biological function of SPX is at the very beginning and what the main function SPX exerts is still unclear. In mammals, SPX was found to participate in inducing stomach contraction^1^, inhibiting adrenocortical cell proliferation^9^, postnatal hyperoxia response^10^, cardiovascular and renal modulation^11^, nociceptive response^12^, fatty acid absorption and weight regulation^13^. In teleosts, functional study of SPX1 mainly focused on its inhibitory role in the regulation of reproduction^4^ and food intake^5,14^.

The biological function of SPX1 in teleosts was preliminarily revealed in goldfish and zebrafish. Most research methods were based on evaluation of the expression level changes during the physiological process or the exogenous administration regulatory effect *in vivo*. To better support or test the conclusion on the function of a newly discovered peptide and to find out more of its potential role *in vivo*, one need to study the gene by loss of function experiment. It is critical to know what happens when the neuropeptide go wrong so that we can fully understand the biological function of it. Therefore, we have produced the *spx1* ^***−/−***^ mutant zebrafish based on transcriptional activator like effector nucleases (TALENs) technique. By assessing the mutant phenotypes especially in regarding with the food intake and energy homeostasis, our results demonstrate that SPX1 might act as a satiety signal for feeding control by suppressing the expression of AgRP1, a well-known and potent orexigenic factor, in zebrafish.

## Results

### Establishment of the zebrafish *spx1*^*−/−*^ mutant lines

**Figure 1:**
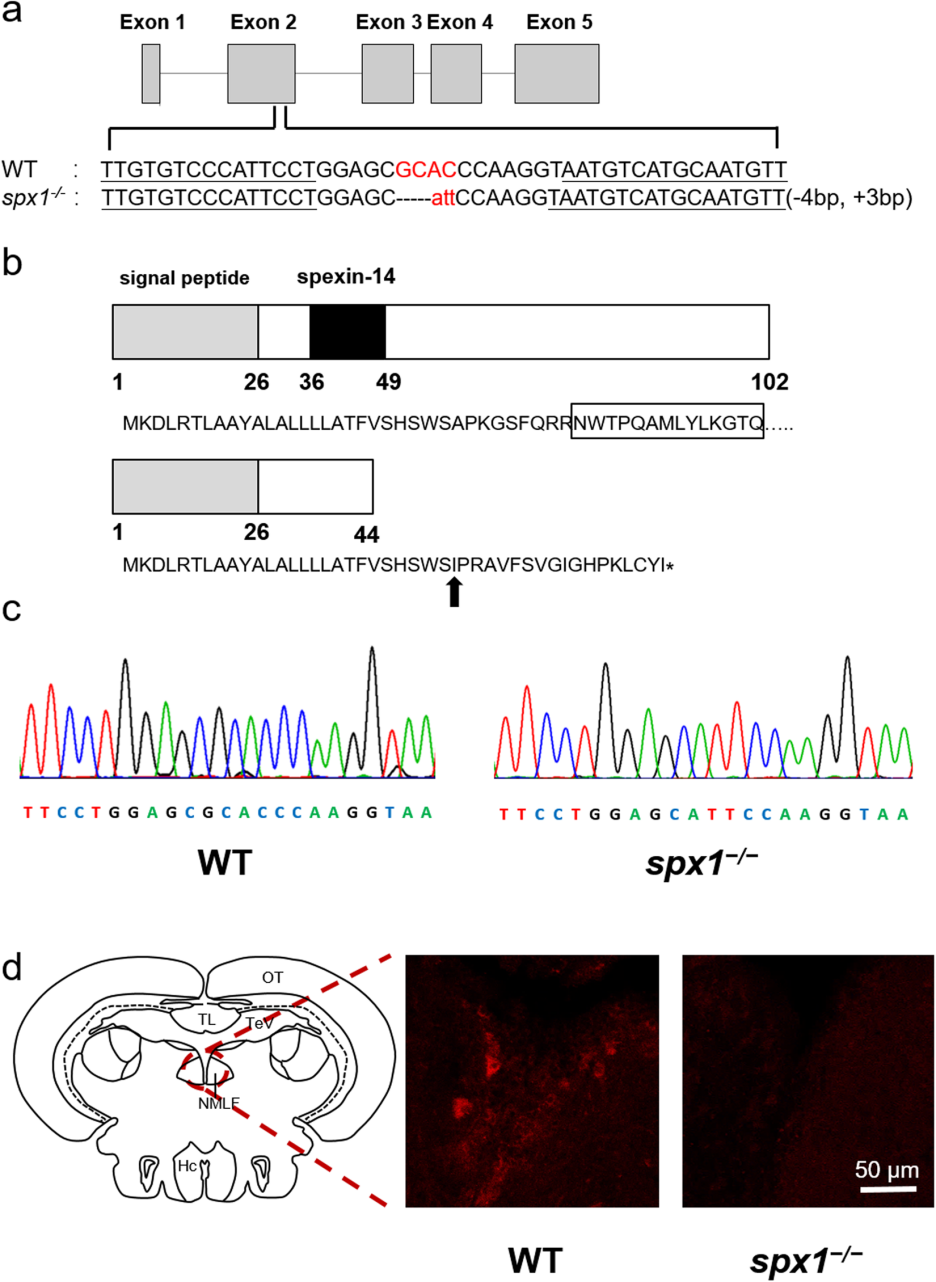
Targeted disruption of zebrafish *SPX1* gene. (**a**) The location of the TALEN binding sites on the zebrafish *SPX* gene and the mutated genotype analyzed in this study. The TALEN binding sites are underlined. The inserted nucleotides are shown in lower case letters. (**b**) Nucleotide and amino acid sequence data for wild type and *spx1* ^***−/−***^ genotype. Letters in box indicates the mature 14 amino acid SPX1 peptide. The new stop codon of the mutant is indicated by asterisk. Black arrow indicates the mutation starting position. (**c**) Comparison of two genotype sequences. (**d**) Detection of SPX1 expression in the brain of WT and *spx1* ^***−/−***^ mutant fish. Note that the expression of SPX1 (red) is present in the nucleus of medial longitudinal fasciculus (NMLF) in WT while no signal can be observed in the mutant one. Hc, caudal zone of the periventricular hypothalamus, NMLF, nucleus of the medial longitudinal fascicle; OT, optic tectum; TeV, tectal ventricle; TL, torus longitudinalis.

After injecting the TALENs mRNA into the embryos, the mixed genomes of the pooled embryos were confirmed to include several kinds of mutation sequence in the SPX1 targeted site (Supplementary Fig. S1). The rest embryos were raised to the adulthood and outcrossed with the WT fish. The heterozygous F1 (*SPX1* ^+*/−*^) with identical mutation sequence, termed three base pairs substitutions and one base pair deletion, were further selected to establish the homozygous mutant line. The mutation could induce open reading frame (ORF) shifts and thus generate truncated proteins with no functional SPX1 mature peptides (Fig. 1a,b). Two genotypes were obtained by sequencing the F2 progeny (Fig. 1c).

The SPX1 protein expression was also detected in the WT fish and mutant line. Immunofluorescent results showed that SPX1 was found in the nucleus of medial longitudinal fasciculus in WT, but no signal was observed in the brain of *spx1* ^*−/−*^ mutant fish (Fig. 1d). In addition, the SPX1 protein expression could not be detected in the ovary of the mutant line as well (Supplementary Fig. S2).

### Gonadal histological examination

**Figure 2:**
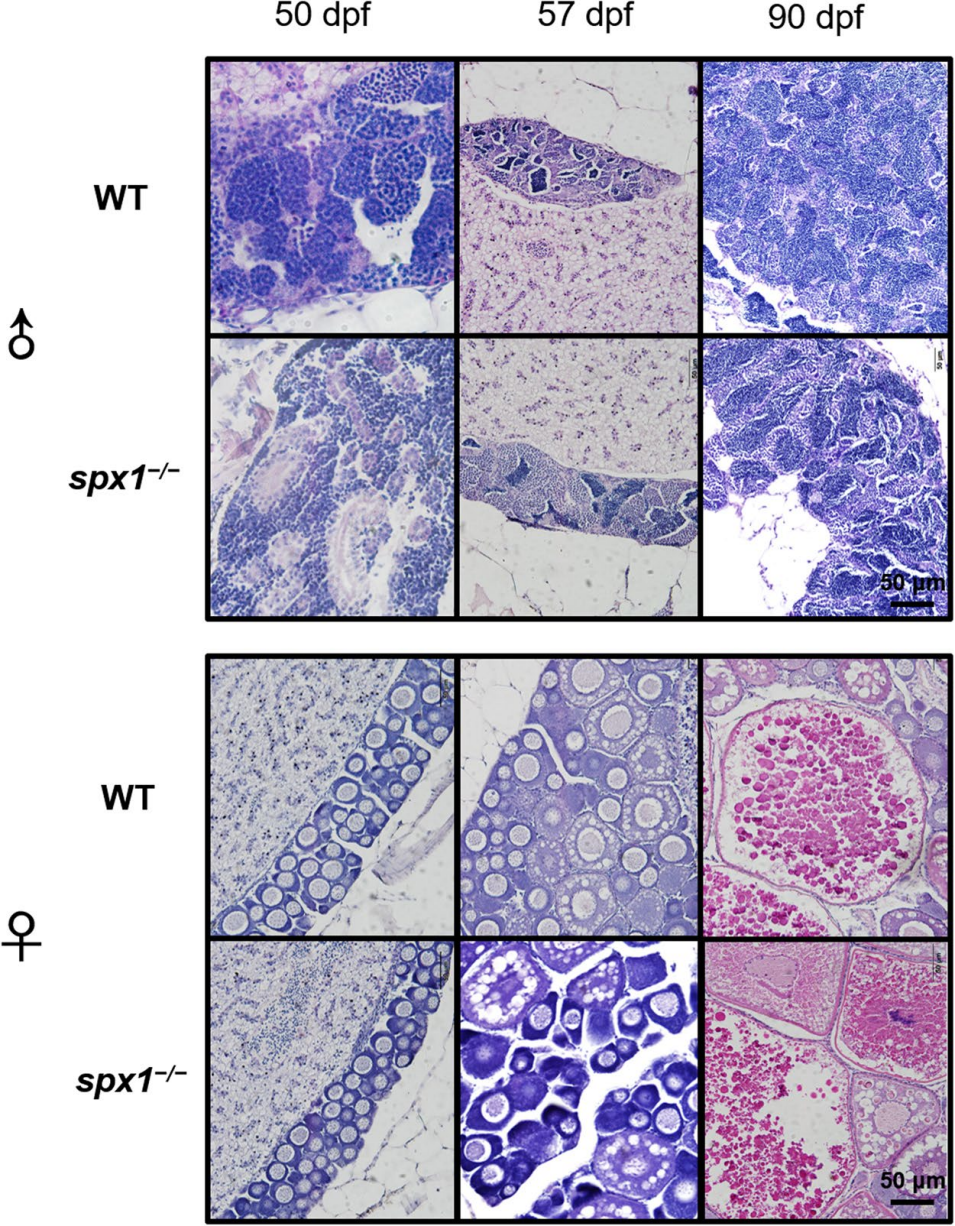
Gonad histology of the zebrafish spx*1* ^−*/*−^ mutant lines during puberty. Male and female zebrafish were sampled at 50, 57 and 90 dpf.

By examining the gonad histology, we found that the deletion of SPX1 did not affect the puberty onset and the gamete maturation. At 50 dpf, spermatids could be observed in both the testis and only the follicles at primary growth (PG) stage could be found in ovaries. At 57 dpf, follicles at previtellogenic (PV) stage began to appear in both the ovaries, suggesting the start of the vitellogenic growth and the puberty onset^15^. At 90 dpf, spermatozoa and fully grown follicles could be observed in the both genotypes (Fig. 2).

### Food intake

**Figure 3:**
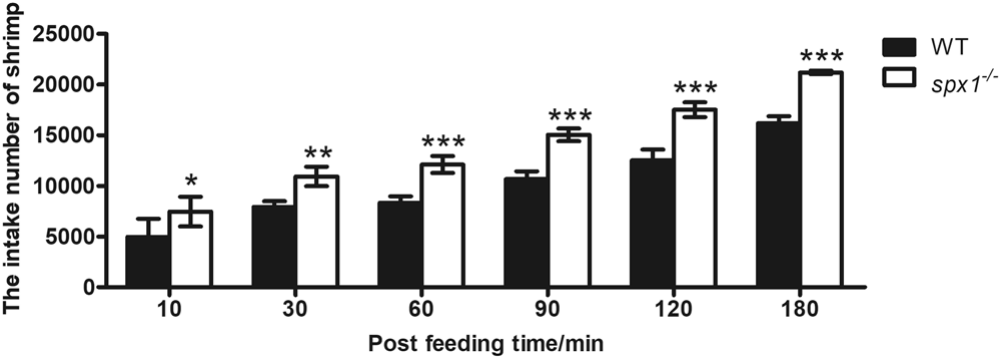
Quantitative analysis of zebrafish food intake using brine shrimp as prey. Data were obtained in triplicate and are represented as mean ± s.e.m. (*n = *3). *P < 0.05; **P < 0.01; ***P < 0.001 by two-way ANOVA with Bonferroni’s *post hoc* test (versus corresponding WT group at each time point).

Six fish with similar body weight were chosen as a group and put in a tank. The zebrafish continued to eat throughout the food intake trial for 3 h and the amount of food consumed was judged by the continuous decline of the shrimp number left in the tanks. It showed that the SPX1 deficient zebrafish had a higher food intake than the WT fish. The number of shrimp consumed by *spx1* ^***−/−***^ fish 3 hours post feeding was about 1.4 times as high as that of the WT ones (Fig. 3).

### mRNA expression of appetite related genes during feeding

**Figure 4:**
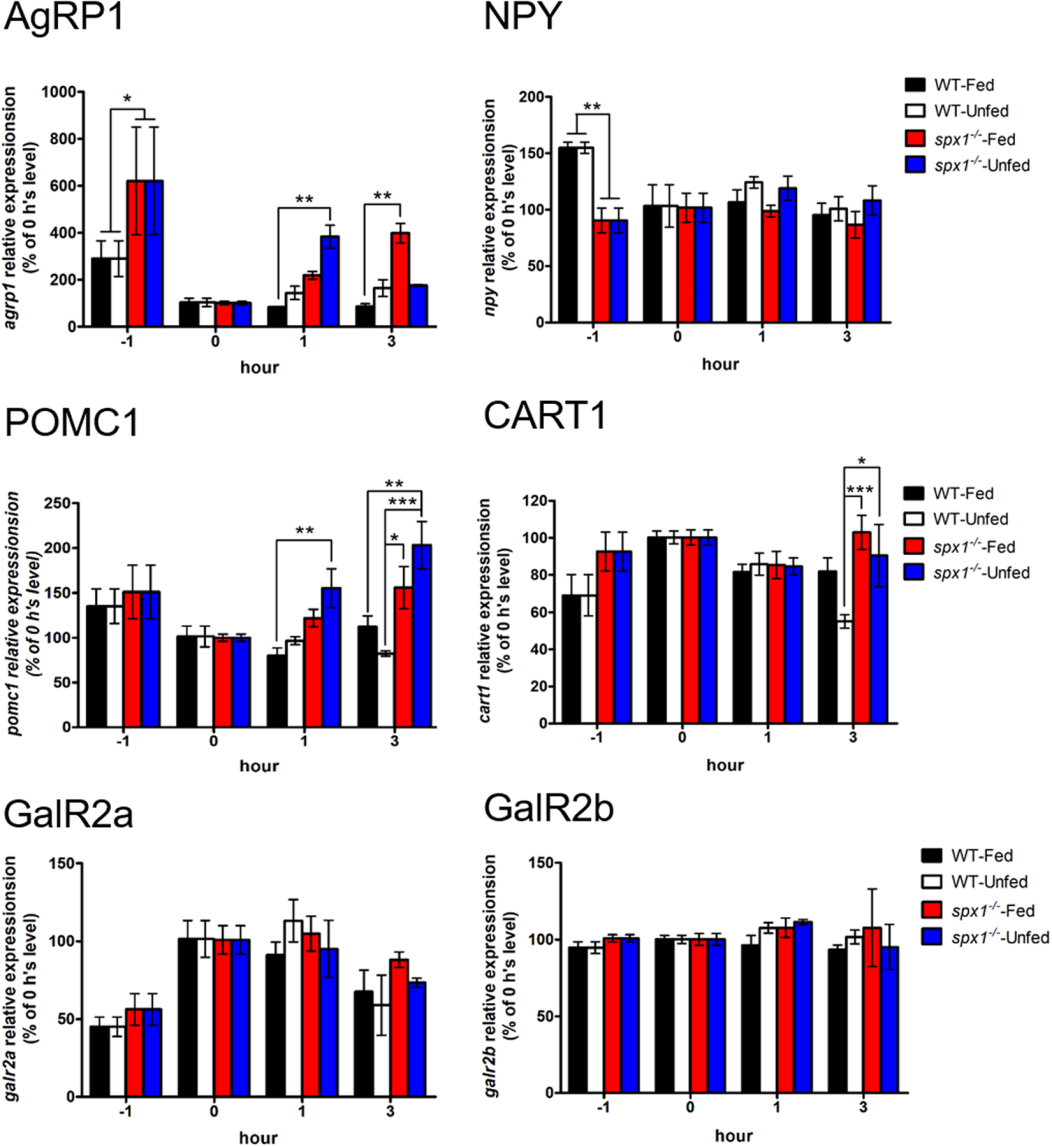
Gene expression analysis of neuropeptides and receptors in the hypothalamus in response to feeding. Quantitative PCR analysis is used to measure mRNA levels of AgRP1 and NPY (upper panel), POMC1 and CART1 (middle panel), GalR2a and GalR2b (lower panel) in WT and *spx1* ^*−/−*^ zebrafish at 1 h, 0 h before feeding, 1 h and 3 h after feeding. Expression of elongation factor 1 alpha is used to normalize all samples. All relative expressions are represented as the ratio to the 0 h level respectively. Data were obtained in triplicate and are represented as mean ± s.e.m. (*n = *3). *P < 0.05; **P < 0.01; ***P < 0.001 by two-way ANOVA with Bonferroni’s *post hoc* test.

Since the *SPX1* knockout fish ate more than the WT ones, we then asked a question: is any neuroendocrine pathway affected by deleting the SPX1? To compare the expression pattern of appetite related genes between the WT and the knockout ones, we examined the several important factors, including two orexigenic factors: Neuropeptide Y (NPY) and agouti-related protein 1 (AgRP1), two anorexigenic factors: proopiomelanocortin 1 (POMC1) and cocaine-amphetamine-regulated transcript 1 (CART1) as well as two receptors of SPX1. We found that the expression pattern during prandial of most appetite related factors were similar between the mutant and WT fish except *AgRP1*. In WT fish, the expression level of *agrp1* seemed not to be affected by a meal and remained rather similar between fed and unfed group, which was consistent with the observation in medaka^16^. However, in the *spx1* ^*−/−*^ fish, the expression level of *agrp1* up-regulated significantly after feeding, and continued to rise even higher than that of unfed group at 3 hours post feeding (Fig. 4).

### mRNA expression of appetite related genes after intracranial administration of SPX1

**Figure 5:**
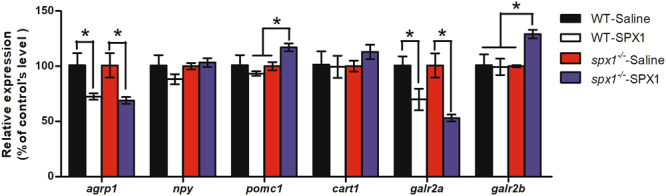
Gene expression analysis of neuropeptides and receptors in the hypothalamus in response to SPX1 intracranial administration. Quantitative PCR analysis is used to measure mRNA levels of WT and *spx1* ^*−/−*^ zebrafish. Expression of elongation factor 1 alpha is used to normalize all samples. All relative expressions are represented as the ratio to the saline control. Values are means ± s.e.m. (*n = *3). *P < 0.05 by one-way ANOVA with Bonferroni’s *post hoc* test.

To clarify whether the expression of *agrp1* was determined directly by SPX1, intracranial administration of SPX1 was performed. In the WT fish, both *agrp1* and *galr2a* down regulated significantly at 1 hour after SPX1 intracranial administration (Fig. 5). Interestingly, the influence of SPX1 administration seemed to be more potent in the *spx1* ^***−/−***^ fish, which were supposed to be unable to synthesize mature SPX1. Not only did the expression of *agrp1* and *galr2a* reduced to half but also *pomc1*, an important anorexigenic factor, and *galr2b* increased significantly (Fig. 5).

### Serum biochemical analyses

**Figure 6:**
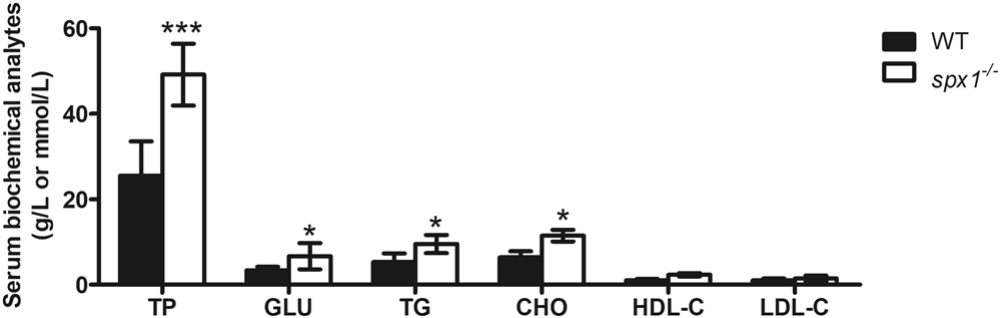
Serum biochemical analytes level in WT and *spx1* ^***−/−***^ zebrafish. For each sample, serum from 10 to 20 fishes was mixed as a pool. Data are represented as means ± s.d. (*n* = 5). *P < 0.05; ***P < 0.001 by Student’s *t* test (versus corresponding WT group at each time point). TP, total protein (g/L); GLU, glucose (mmol/L); TG, triacylglycerol (mmol/L); CHO, cholesterol (mmol/L); HDL-C, high density lipoprotein cholesterol (mmol/L); LDL-C, low density lipoprotein cholesterol (mmol/L).

Blood was collected from both the WT and *spx1* ^***−/−***^ fish to analyze the nutrition concentration. To avoid the influence of the acute feeding, blood collection was performed after fasting for 1 day. The serum concentration of glucose, triacylglycerol and cholesterol of *spx1* ^*−/−*^ mutant fish were all found to be significantly higher than that of the WT one (Fig. 6).

### Growth rate and body fat percentage

**Figure 7:**
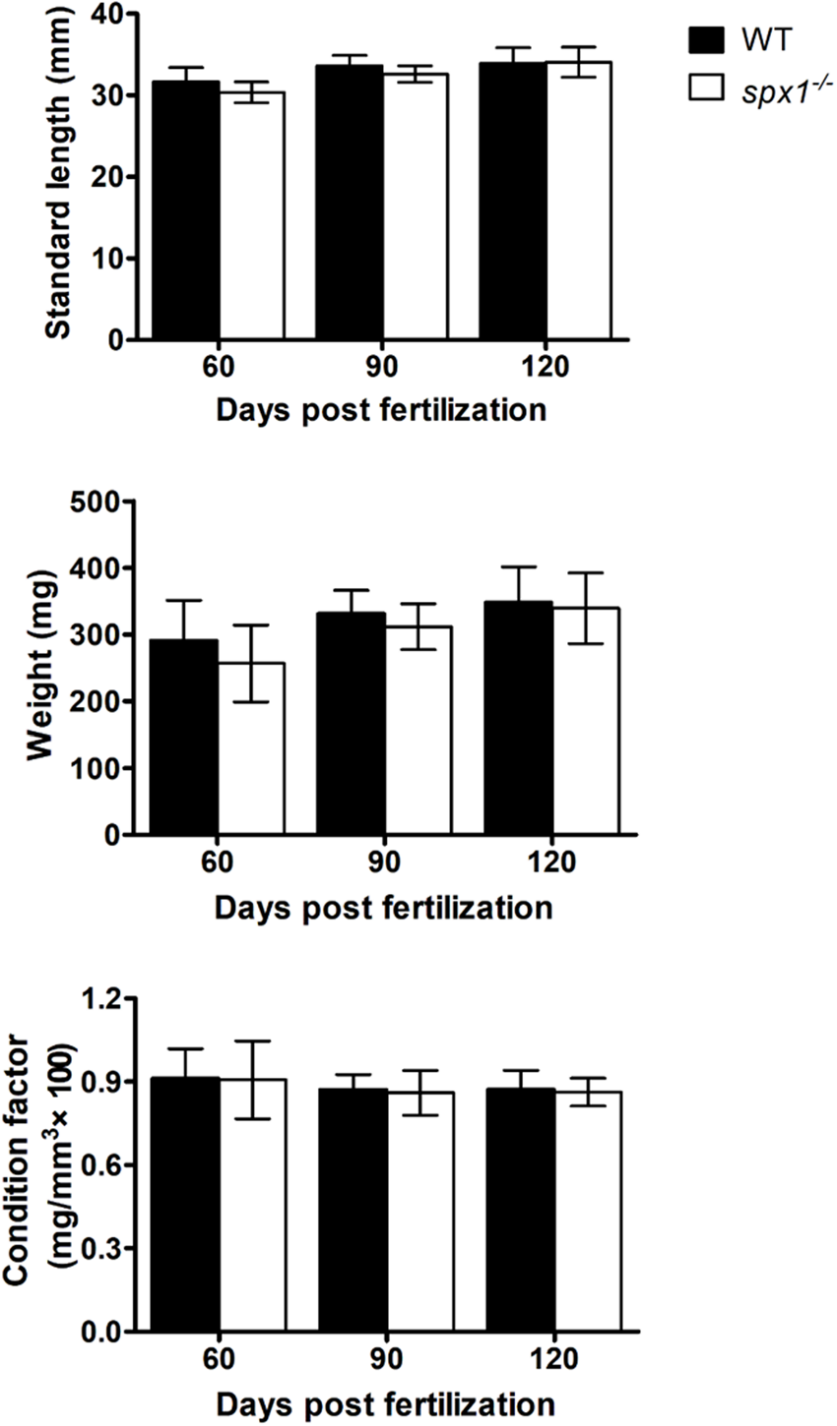
The standard length, body weight and the condition factor of WT and *spx1* ^***−/−***^ zebrafish on different day post fertilization. Two-way ANOVA was performed and difference was not significant. Data are represented as means ± s.d. (*n = *8).

By measuring the growth rate of WT and *spx1* ^*−/−*^ zebrafish on different day post fertilization, we found that there were no statistically significant differences in body weight (BW), standard length (SL) and conditional factor between the two groups (Fig. 7). No significant difference of body fat percentage between two groups was observed either (WT: 22.22 ± 3.01% versus *spx1* ^−/−^: 19.84 ± 2.39%, *n* = 6, mean ± s.e.m.). The hyperphagia seemed to have no stimulatory effect on growth of *spx1* ^*−/−*^ zebrafish.

## Discussion

To explore the biological function of SPX1 in teleost, we targeted to disrupt the sequence of *SPX1* in zebrafish using our optimized TALENs system^17–19^. The mutation was three base pairs substitutions and one base pair deletion, inducing the ORF shift and a new stop codon right at the position of the mature peptide sequence. We have further demonstrated that SPX protein is absent in the *SPX1* mutant line, indicating that *SPX1* mutant zebrafish lines were successfully established.

In our previous study, SPX1 mature peptide could suppress the LH release in goldfish, acting as a negative regulator of hypothalamic–pituitary–gonadal axis^4^. However, no difference was found on the gonadal development between WT fish and *SPX1* mutant fish. The puberty onset and gamete maturation are not affected in the absence of functional *SPX1* genes. These data indicate that SPX are not essential for zebrafish reproduction. It is understandable that no significant reproductive phenotype was observed after *SPX1* knockout in zebrafish. It is proposed that teleost fish have evolved a complex neuroendcrine system to control the reproductive axis, which is likely regulated by multiple independent neuroendocrine factors^20–22^. The loss of one neuropeptide in reproductive axis could be compensated by other neuropeptides. For example, *kiss*/*kissr* gene knockout does not impair zebrafish reproductive functions^20^.

Using the *SPX1* knockout fish, we found that zebrafish might possess an appetite-signaling pathway in the hypothalamus that is related to SPX1. This is based on the observed difference in mRNA expression of AgRP1 during a meal and an observed increase in food intake in *spx1* ^*−/−*^ mutant fish.

By selecting genes that have already been shown to play key roles in food intake and as such could serve as indicators of anorexigenic and orexigenic function and comparing the expression pattern of these genes during the periprandial between the *spx1* ^*−/−*^ mutant fish and WT one, we found that *agrp1* was significantly upregulated in the *spx1* ^*−/−*^ mutant fish at 3 h post feeding. To further confirm the relation between SPX1 and AgRP1, we performed the intracranial administration of SPX1 to the fish. An hour after the administration, we found that the *agrp1* was significantly down regulated in the hypothalamus of both WT and *spx1* ^*−/−*^ fish. In the mean while, the expression level of *pomc1* and *galr2b* in *spx1* ^*−/−*^ group was significantly increased compared to the WT and saline administration group. The deletion of SPX1 seemed to increase the response sensitivity of the anorexigenic *pomc1* and the SPX receptor *galr2b* to the SPX1 stimulation. Correspondingly, we have observed that the food intake of *spx1* ^*−/−*^ mutant zebrafish was higher than that of WT fish. Evidence above was consistent with the previous discovery in goldfish^5^. In zebrafish, the overexpression of *agrp* could promote the food intake and increase the fat accumulation^23^, indicating AgRP as an important orexigenic factor in energy homeostasis. Therefore, it is reasonable to conclude that SPX1 serves as a satiety signal by suppressing the expression of orexigenic *agrp1*. In zebrafish. SPX1 expression neurons were identified in the NMLF. Further study on the co-localization of the receptors of SPX1, GalR2a/b and AgRP1 in hypothalamus is needed to support the hypothesis.

The hyperphagia did lead to a higher serum concentration of various kinds of nutrition including glucose, triacylglycerol, cholesterol and high density lipoprotein cholesterol (HDL-C) in *spx1* ^*−/−*^ mutant fish. High level of glucose and triacylglycerol in blood is considered as indicators of overnutrition, which can be resulted from hyperphagia. In contrast, the main function of HDL-C is to transport the excess cholesterol in tissues and blood vessels back to the liver to metabolite and excrete, which can prevent obesity^24^. It seems that the deletion of *SPX1* in zebrafish induced the hyperphagia and higher nutritional level as well as higher metabolism level. However, we could not find any differences of the growth rate including the standard length, body weight or condition factor on different day post fertilization between the *spx1* ^*−/−*^ mutant and the WT fish. The hyperphagia could not promote the growth of zebrafish. In medaka, the leptin-receptor deficient models also exhibited a higher food intake with normal growth rate in adult fish^16^. Since the mechanism controlling the energy homeostasis is far too complex, only increasing food intake might not definitely accelerate the growth rate. The efficiency of energy absorption and conversion as well as individual metabolism level might altogether determine the growth rate.

In summary, we have generated zebrafish mutant lines lacking the *SPX1* gene. We have found that the deletion of SPX1 in zebrafish had no effect on puberty onset or gamete maturation, but could induce the elevated orexigenic *agrp* expression and the increased amount of food intake. The hyperphagia led to higher nutritional serum level but could not stimulate the growth rate. Our study demonstrated that SPX1 controlled the food intake as a satiety signal in the energy homeostasis. To our best knowledge, this is the first study to generate *SPX* knockout animal models in vertebrates and characterize the phenotype involved. Moreover, the mutant fish line generated in this study would be powerful models to investigate *SPX1* functions in the regulation of other biological processes.

## Materials and Methods

### Fish husbandry

AB strain and *spx1* ^***−/−***^ zebrafish were maintained in flow-through aquaria under an artificial photoperiod of 12 hour light and 12 hour dark at 28 °C. Fish were fed twice a day, at 10:00 am and 16:00 pm respectively, with brine shrimp and commercial fish food pellet.

All animal experiments were conducted in accordance with the guidelines and approval of the respective Animal Research and Ethics Committees of the Sun Yat-Sen University and the Chinese University of Hong Kong.

### Transcription activator-like effector nucleases (TALENs) preparation

The TALENs were assembled using the golden gate method as described previously^17–19^. Briefly, TALENs were assembled by 2 digestion-ligation steps. In the first round of digestion-ligation, modular plasmids recognizing each nucleotide were digested and ligated into the backbones of 2 middle array plasmids. In the second round, the middle array plasmids and the last repeat plasmid were cloned into the backbones of 2 optimized TALEN expression plasmids (the pCS2-TALEN-ELD and pCS2-TALEN-KKR) developed by our group^17,18^. The final TALEN expression plasmids were linearized by *Not* I restriction enzyme digestion. TALEN mRNAs were transcribed using the mMESSAGE mMACHINE SP6 kit (Ambion, USA) and purified using the RNeasy Mini kit (QIAGEN, Germany).

### Establishment of zebrafish mutant lines

The *in vitro* transcribed TALEN mRNAs (200–500 pg) were microinjected into 1-cell stage zebrafish embryos. After two days, genomic DNA was isolated from 10 pooled fertilized embryos. The target genomic regions were amplified by PCR and subcloned into the pTZ57R/T vector (Fermentas, USA). Single colonies were genotyped by sequencing. Once the mutation was confirmed in the pooled embryos, the rest ones were raised to adulthood and outcrossed with WT fish. The heterozygous F1 progeny were genotyped by sequencing the genomic DNA from the cut tail fin and those with the same mutation sequence were obtained and self-crossed. And finally about a quarter of the F2 progeny obtained were homozygous mutants. The primers used in this study were listed in Supplementary Table S1.

### Gonad histological examination

Under our lab’s aquarium condition, the puberty onset of zebrafish start at around 57 days post fertilization (dpf) when the body weight and length reach over 100 mg and 1.80 cm, respectively^25^. To examine the gonad development in different stages, fish at 50, 57 and 90 dpf were euthanized and then decapitated. The decapitated fish was immediately fixed in Bouin’s solution at room temperature for 24 hours. The fixed samples were dehydrated through a graded series of ethanol and embedded in paraffin wax and then cut into 7-μm sections. After rehydration, slides were stained with hematoxylin and eosin.

### Immunofluorescence (IF) of zebrafish SPX1

The whole brain or ovary of zebrafish was fixed with 4% paraformaldehyde in PBS (pH 7.4) at 4 °C overnight, and then dehydrated in 30% sucrose in PBS at 4 °C for 24 hours. The samples were embedded in Tissue-Tek OCT compound (Sakure, Germany) and sectioned serially at 7 μm. Sections were blocked with 1% Triton × 100 normal goat serum at room temperature for 1 hour. Primary rabbit polyclonal antibody against human spexin-14a (Phoenix Pharmaceuticals, China) was diluted at 1:100 and incubated with sections at 4 °C overnight. After washing several times, sections were incubated with the 1:200 diluted goat anti-rabbit IgG conjugated Cy3 or fluorescein FITC (Sigma, USA) in dark. And then the sections were mounted and visualized with Laser Scanning Confocal Microscope (Leica TSC SP5, Germany).

### Quantitative real-time PCR (qRT-PCR)

Total RNAs were isolated from different tissues and three or four samples were pooled together if necessary. One microgram of total RNA was reverse transcribed into cDNA using the ReverTra Ace-α first-strand cDNA Synthesis kit (TOYOBO, Japan). qRT-PCR was performed on a Roche LightCycler 480 real-time PCR system using Realtime SYBR Green I PCR Master Mix (TOYOBO, Japan). Reaction conditions were as follows: denaturation at 95 °C for 1 minute, followed by 40 cycles of 95 °C for 15 seconds, 56 °C for 15 seconds, 72 °C for 20 seconds.

### Food intake

Measures to quantify food intake of zebrafish was performed using the method described previously^16^ with slight modifications. Briefly, six WT fish (276.1 ± 16.6 mg) and six SPX1 mutant fish (275.0 ± 16.9 mg) were stocked in a 2-L tank, respectively. Shrimp (1500 shrimp/100 mg BW) were added to the tanks at normal feeding time (10:00). The number of shrimp remaining in the tank were counted at 10, 30, 60, 90, 120 and 180 min after feeding. The total number of shrimp in the tank was calculated according to the counting number of the 25-ml aliquot water.

### Post-prandial expression of appetite-related genes during feeding

Adult WT and SPX1 mutant zebrafish were fed daily at a scheduled time (10:00 am) under the same conditions. The daily scheduled feeding time was taken as time zero (0 h). The hypothalamuses were sampled at the following feeding times (−3 h, 0 h, 1 h, 3 h).

### Intracranial administration of SPX1

Intracranial administration was performed using the method described previously^26^. Briefly, anesthetized fish were placed on a sponge soaked with water containing 0.05% MS222, and the skulls were impaled with a 0.36 mm diameter needle of the syringe in the midline at the telencephalon–diencephalon border. The fish were intracranially injected with 1 µL of either saline or SPX1 (10 pmol/µL in saline, NWTPQAMLYLKGAQ-NH2, purity > 95%, GL Biochem, China) into the cranial cavity by a heat-pulled glass capillary micropipette attached with a microinjector. One hour after the administration, hypothalamus were sampled and three hypothalamus were mixed as a pool to yield enough total RNA.

### Blood collection

Blood collection was performed using the method described previously^27^ with slight modifications. Briefly, the fish were placed into the ice water until it get completely anesthetized. Cut the fish between the anal fin and the caudal fin to make a diagonal incision and immediately after the blood come out, aspire the blood with a glass capillary tube (0.7 mm in diameter) until the blood stop. Gently transfer the aspired blood into a tube and centrifuge for 10 minutes at 400 g at 4 °C. Aspirate the serum with a pipette from the top layer of the tube. Mix the serum from different fish if necessary.

### Body fat percentage measurement

Zebrafish were euthanized with 0.3% MS222 and the wet body weight (W_W_) was measured. Fish was cut into small pieces and then put into the mortar containing liquid nitrogen, followed by grinding completely into powder. Powder was put into oven at 80 °C overnight to dehydrate. The dry powder was measured as dry body weight (W_D_). Lipids in the powder was extracted by Soxhlet extraction methods using petroleum ether as solvent. After removing the lipid, the weight of the remaining powder was measured as W. The body fat percentage (BFP) can be calculated as follow: BFP = (W_D_ − W)/W_W_ × 100%.

### Statistical analyses

Statistical analyses were performed using one-way ANOVA followed by Bonferroni’s *post hoc* test to compare the neuropeptide response to SPX1 administration (Fig. 5); Two-way ANOVA followed by Bonferroni’s *post hoc* test to compare the consumed number of shrimp (Fig. 3) and neuropeptide expression level (Fig. 4) during a meal among different groups across different time points and to compare the growth rate on different day post fertilization (Fig. 7); Student’s *t* test to compare serum analytes level (Fig. 6) and body fat percentage between the WT and mutant group. *P* < 0.05 was considered statistically significant.

### Data availability

Data supporting the findings of this study are available in the article and its Supplementary Information files, or from the corresponding authors on reasonable request.

## Electronic supplementary material

Supplementary Information
