# Supplementary material for: Spexin Suppress Food Intake in Zebrafish: Evidence from Gene Knockout Study
Source: Sci Rep. 2017 Nov 7;7:14643. doi: 10.1038/s41598-017-15138-6 (PMC5677112; doi:10.1038/s41598-017-15138-6)
Supplement: Supplementary file 1 — Supplementary Information [file 41598_2017_15138_MOESM1_ESM.pdf]

Spexin Suppress Food Intake in Zebrafish: Evidence from Gene Knockout Study

Binbin Zheng[1, +], Shuisheng Li[1, +], Yun Liu[1], Yu Li[1], Huapu Chen[1], Haipei Tang[1], Xiaochun Liu[1], Haoran Lin[1§], Yong Zhang[1, *], Christopher H.K. Cheng[2, *]

[1] State Key Laboratory of Biocontrol, Institute of Aquatic Economic Animals, and the Guangdong Province Key Laboratory for Aquatic Economic Animals, School of Life Sciences, Sun Yat-Sen University, Guangzhou 510275, China.

[2] School of Biomedical Sciences, The Chinese University of Hong Kong, Shatin, New Territories, Hong Kong, China.

***Corresponding authors**:

Yong Zhang, State Key Laboratory of Biocontrol, Institute of Aquatic Economic Animals, and the Guangdong Province Key Laboratory for Aquatic Economic Animals, School of Life Sciences, Sun Yat-Sen University, Guangzhou 510275, China. Tel: +86-20-84110188; Fax: +86-20-84113717; E-mail: lsszy@mail.sysu.edu.cn

Christopher H.K. Cheng, Rm 604A, 6/F., Lo Kwee Seong Integrated Biomedical Sciences Building, The Chinese University of Hong Kong, Shatin, N.T., Hong Kong, China. Tel: +852-39436801; E-mail: chkcheng@cuhk.edu.hk

+ These authors contributed equally to this work.

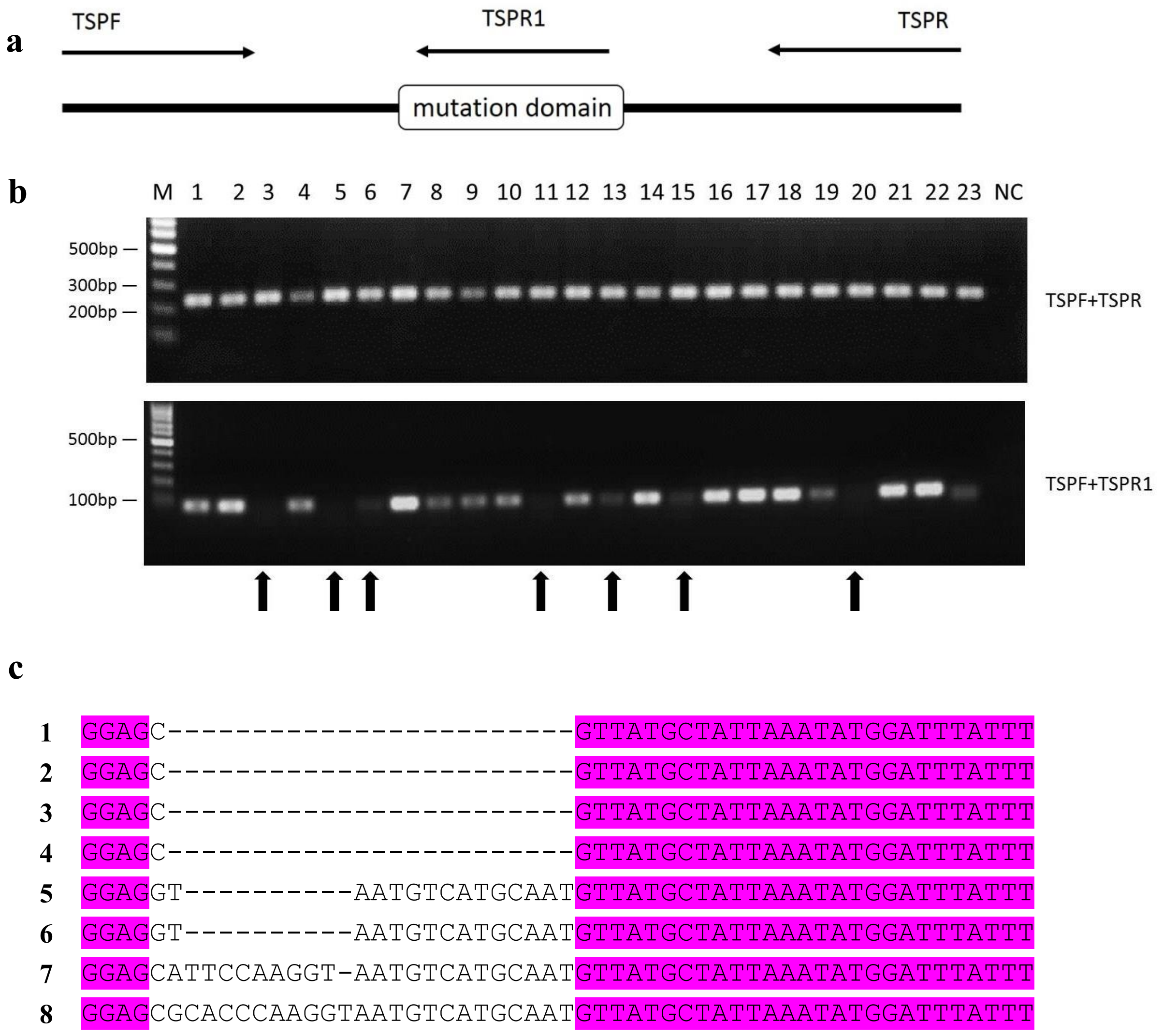

Supplementary Fig. S1. Mutagenesis detection on P0 zebrafish by PCR. a: Schematic drawing of primer pairs used for detecting mutagenesis. b: DNA gel electrophoresis of colony PCR with the two primer pairs in fertilized eggs produced by P0 fishes outcrossing with wild type ones. Arrows indicate mutation colonies. NC: negative control with no template added. c: Sequence alignment of mutated single colonies. Number 1 to 7 are mutation sequences. Number 8 is the wild type sequence.

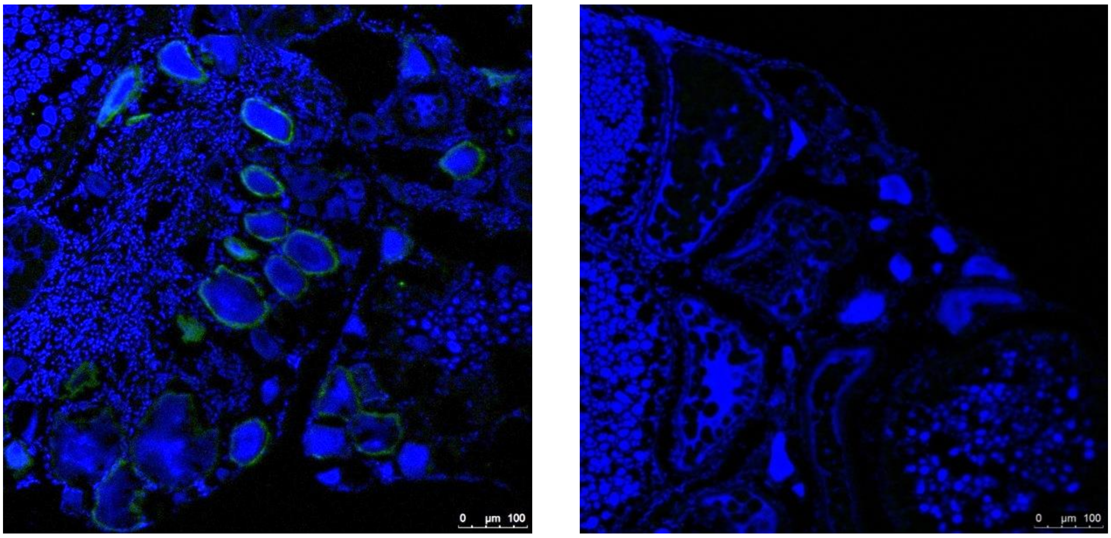

WT *spx1*$^{-/-}$

Supplementary Fig. S2. Detection of SPX1 expression in zebrafish ovaries of wild type and *spx1*$^{-/-}$ mutant. Note that the expression of SPX1 (green) is present in the wild type ovary while no signal can be observed in the mutant one. Nucleus is stained by DAPI (blue).

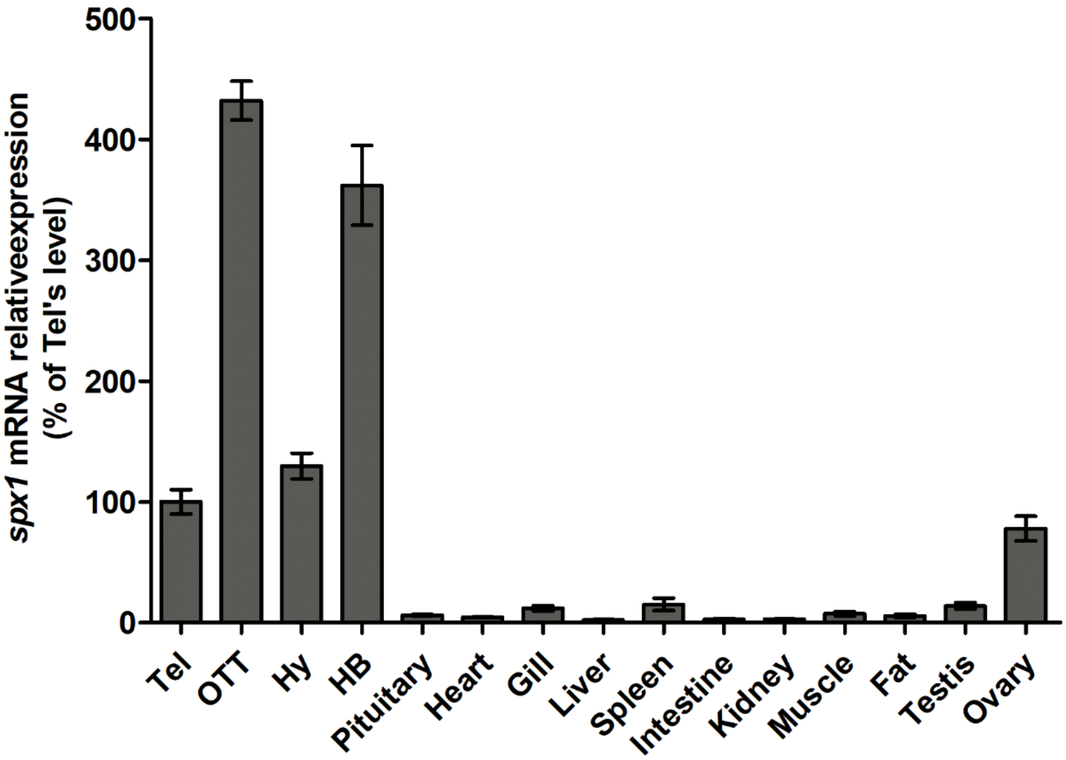

Supplementary Fig. S3. Quantitative real-time PCR analysis of *spx1* mRNA levels in different zebrafish brain regions and tissues. mRNA level from different tissues were normalized against *ef1α* transcripts. Data are expressed as percentages of the Tel group and presented as the mean values ± s.e.m. ($n = 3$). Tel: telencephalon; OTT: optic tectum thalamus; Hy: hypothalamus; HB: hindbrain.

Supplementary Table S1. Primers used in this study

Primer for screening the mutations	Sequences (from 5' to 3')		
TSPF	AGGACTCTTGCGGCGTACGCAC		
TSPR	GCATAATAGGCTATACCATAAC		
TSPR1	CATTACCTTGGGTGCGCTCCAG		
Primer for realtime PCR	Sequences (from 5' to 3')	PCR efficiency	Genebank accession No.
NPY F	CGCTGACACCTTAATTTCAGACC	100.4%	NM_131074.2
NPY R	TGGATGAGATCACCATGCCAA		
AgRP1 F	AGACCTTGAAGCCTATGATGAG	94.8%	XM_009303347.1
AgRP1 R	GCCTTAAAGAAGCGGCAGTA		
POMC1 F	CCCCCTACAAAATGACCCAT	101.7%	NM_181438.3
POMC1 R	ATCCTTCCTCGGTTGGTCTT		
CART1 F	CCTGCAGCTTCTCCATCCTC	104.1%	GU057833.2
CART1 R	GGTAAACAACACACTGGAGCATT		
GalR2a F	TCCAATCATTGCGACGGGTCA	95.0%	XM_002664007.3
GalR2a R	AATGTCATAAAGGCGCTCGTCA		
GalR2b F	ACGGCTAATGATGCCTTACAGA	95.0%	XM_001339133.3
GalR2b R	GTGCTCCTTCAGTCAATGCAGA		
eF1α F	GCTCGTTTTGAGGAAATCACC	94.5%	BC060907.1
eF1α R	CCATCCTGAAATTGGGACGAA		
